# The role of endoplasmic reticulum-mitochondria contact sites in the control of glucose homeostasis: an update

**DOI:** 10.1038/s41419-018-0416-1

**Published:** 2018-03-09

**Authors:** Jennifer Rieusset

**Affiliations:** Laboratoire CarMeN, Unité Mixte de Recherche INSERM U-1060 et INRA U-1397, Université Lyon 1, Oullins, 69600 France

## Abstract

The contact sites that the endoplasmic reticulum (ER) forms with mitochondria, called mitochondria-associated membranes (MAMs), are a hot topic in biological research, and both their molecular determinants and their numerous roles in several signaling pathways are is continuously evolving. MAMs allow the exchange between both organelles of lipids, calcium (Ca^2+^), and likely reactive oxygen species, allowing adaptations of both cellular bioenergetics and cell fate depending of cellular needs or stresses. Therefore, it is not surprising that MAMs affect cellular metabolism. Nevertheless, recent arguments suggest that MAMs could also act as key hub of hormonal and/or nutrient signaling in several insulin-sensitive tissues, pointing a specific role of MAMs in the control of glucose homeostasis. Here, I provide a brief review and update on current key signaling roles of the MAMs in the control of glucose homeostasis in both health and metabolic diseases. Particularly, the relevance of ER-mitochondria miscommunication in the disruption of glucose homeostasis is analyzed in details in the liver, skeletal muscle, adipose tissue, and beta cells of the pancreas.

## Facts

- ER-mitochondria contact sites are highly dynamic structures controlling lipid and calcium homeostasis, mitochondria metabolism, as well as several intracellular processes and signaling pathways.
- ER-mitochondria contact sites are new hubs of insulin and/or glucose signaling in several peripheral tissues.
- ER-mitochondria communication plays a key role in the control glucose homeostasis.
- ER-mitochondria miscommunication is associated with metabolic diseases.

## Open questions

- How is regulated ER-mitochondria tethering?
- How MAMs control insulin signaling in peripheral tissues?
- Does MAM integrity control insulin secretion in beta cells of pancreas?
- Unravelling the controversy on the extent of ER-mitochondria interactions in hepatic insulin resistance.
- Is ER-mitochondria miscommunication a cause or a consequence of metabolic diseases?

## Introduction

**Fig. 1:**
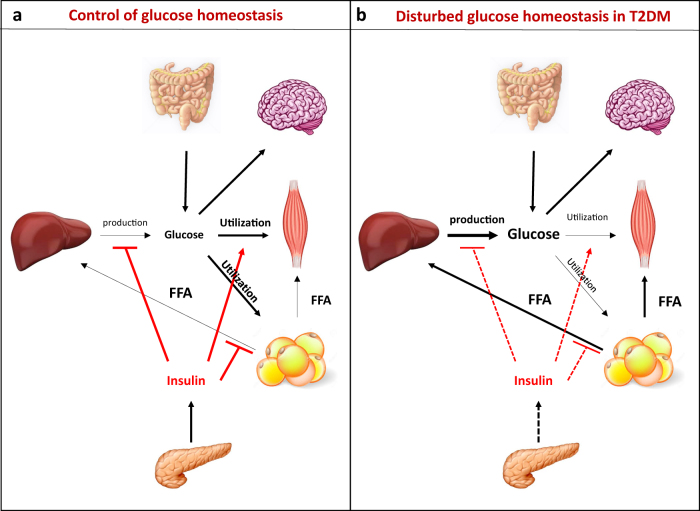
Tissue interplay in the control of glucose homeostasis. **a** After a meal, the glucose is absorbed by the gut and the increase of blood glucose levels stimulates the secretion of insulin by beta cells of the pancreas. Then insulin inhibits hepatic glucose production and stimulates glucose uptake and utilization by skeletal muscle and adipose tissue in order to maintain systemic glycemia within a physiological range. Insulin also inhibits FFA release by adipose tissue in order to favor glucose metabolism. **b** Disruption of glucose homeostasis in T2DM, where both insulin resistance and altered insulin secretion by beta cells of the pancreas participate to chronic hyperglycemia

Glucose is an essential nutrient of all mammalian cells, which represents an important source of energy and biomolecule precursor for most tissues. Glucose in plasma either comes from dietary sources or is either the result of the breakdown of glycogen in liver (glycogenolysis) or the synthesis of glucose in liver, kidney or intestine (gluconeogenesis). Therefore, depending of fed/fasted cycles, glucose is either used or produced by the organism in order to maintain systemic glucose levels in a physiological range (4–6 mM), a process called glucose homeostasis. This process is crucial in mammalians because some tissues, such as brain and red blood cells, rely on glucose as the sole energy source. Consequently, several mechanisms have evolved to closely monitor glucose and maintain it in a narrow physiological range, through an intricate regulatory and counter-regulatory neuro-hormonal system^1^. At the tissue level, several organs are involved in the control of glucose homeostasis, and their metabolism (glucose intake, storage, mobilization or breakdown) are orchestrated mainly via the secretion of hormones, like insulin and glucagon. Following a meal, the increase of circulating glucose levels induces insulin secretion in pancreatic β-cells. Then, insulin controls glucose homeostasis by inhibiting hepatic glucose production and stimulating glucose utilization by skeletal muscle and white adipose tissue (WAT). In addition, insulin inhibits triglyceride hydrolysis in WAT, preventing the liberation of circulating free fatty acids (FFA) (Fig. 1a). At the opposite, during fasting periods, glucagon secretion by pancreatic α-cells stimulates gluconeogenesis and glycogenolysis leading to hepatic glucose release.

Disruption of glucose homeostasis underlies the physiopathology of type 2 diabetes mellitus (T2DM), which results from both peripheral insulin resistance and altered insulin secretion by pancreatic β-cells. Insulin resistance is defined as the failure of cells to respond normally to insulin’s glucose-lowering effects. In skeletal muscle, insulin resistance induces a reduction of glucose uptake^2^ whereas, in the liver, altered insulin signalling leads to greater glycogenolysis and gluconeogenesis^3^, thereby raising blood glucose levels. In WAT, insulin resistance leads to increased lipolysis, resulting in elevated circulating FFA levels^4^. Increased FFA levels favor ectopic lipid accumulation in liver and skeletal muscle, further exacerbating insulin resistance, and induce hepatic triglyceride synthesis favouring hyperlipidemia^5^. In order to compensate peripheral insulin resistance, pancreatic β-cells secrete more and more insulin leading progressively to an exhaustion of the pancreas. Therefore, disruption of glucose-stimulated insulin secretion by β-cells ultimately leads to disrupted glucose homeostasis (Fig. 1b).

Molecular mechanisms of both insulin resistance and beta cell dysfunction in T2DM have been intensively studied during the last decades, but are still not fully elucidated. Nevertheless, alterations of both insulin action and secretion are associated with mitochondrial and endoplasmic reticulum (ER) stresses^6–10^. Importantly, ER and mitochondria are no longer considered as individual organelles in the cell, as they are structurally and functionally linked through contact points defined as mitochondria-associated membranes (MAMs)^11–14^. Recent evidences suggest that MAMs could be an important hub for hormonal and/or nutrient signalling in peripheral tissues, such as the liver^15,16^ and skeletal muscle^17^. In addition, ER-mitochondria miscommunication is associated with both hepatic^15,16^ and skeletal muscle^17^ insulin resistance in several models, and with β-cell dysfunction in pancreas of type 2 diabetic patients^18^, highlighting the importance of MAMs in the control of glucose homeostasis. Consequently, targeting MAM structure and function might be a new and interesting strategy to improve glucose homeostasis in T2DM.

In this review, I provide a review and update on the emerging role of ER-mitochondria communication in the control of glucose homeostasis, either directly by regulating insulin and/or glucose signalling pathways or indirectly by influencing organelle homeostasis and several metabolism-related cellular processes. Then, I discuss some evidences pointing a key role of ER-mitochondria miscommunication in the disruption of glucose homeostasis. Compared to precedent reviews on this topic^19–22^ which mainly focused on MAMs in the liver, this one sheds light on new literature concerning skeletal muscle, adipose tissue and beta cells of pancreas, giving a wider view of the role of MAMs in several tissues involved in the control of glucose homeostasis.

## ER-mitochondria communication in the control of glucose homeostasis

Inter-organelle communication is an emerging aspect of cell biology, and the dynamic nature of this networks allows the adaptations of metabolism to cellular needs or stresses. Particularly, the structural and functional interactions between ER and mitochondria play a central role in multiple pathways, ranging from the control of organelle homeostasis to several cellular processes or signalling pathways, all finally impacting cellular metabolism. Therefore, MAMs are progressively emerging as a complex hub fundamental for cellular metabolism, and in particular for glucose homeostasis. MAMs can affect glucose homeostasis either indirectly by modulating mitochondria and ER biology, as well as autophagy, inflammation and immune signalling pathways, which are known to impact metabolism in several metabolic tissues, or directly by modulating nutrient and hormone signalling pathways in insulin-sensitive tissues. Therefore, in the following sections, I briefly present ER-mitochondria signalling through lipid, calcium (Ca^2+^) and reactive oxygen species (ROS) exchange, and further discuss the abundant new literature pointing an emerging and multifaceted role of MAMs in the control of glucose homeostasis.

### Inter-organelle signalling

#### Lipid exchange

The first described function of ER-mitochondria contact sites was exchange of PL between organelles^23^. Indeed, phosphatidylserine (PS) is synthesized in ER by the exchange of serine for the choline or ethanolamine head-groups of phosphatidylcholines (PC) or phosphatidylethanolamines (PE) by PS synthase-1 and PS synthase−2, which are enriched at MAMs^24^. Then, newly-made PS is transferred into mitochondria through MAMs, where it is decarboxylated to PE via PS decarboxylase in mitochondrial inner membrane^25^. PS transfer at MAM interface is mediated by oxysterol-binding proteins-related protein 5 (ORP5) and ORP8, which were also localized at MAMs^26^. PE is also produced at MAMs by acylation of lyso-PE by lyso-PE acyltransferase. Lastly, PE returns to ER, where PE N-methyltransferase methylates it for synthesis of PC. In addition, MAMs can also contain enzymes required for cholesterol and ceramide biosynthesis^27^. Lastly, MAMs have a distinct lipid composition since they are enriched in cholesterol and sphingolipids, making detergent-resistant microdomains at the ER surface^28^. In agreement, caveolin 1 was recently identified at MAM interface where it regulates ER-mitochondria cholesterol transfer^29^.

#### Ca^2+^ transfer

Ca^2+^ uptake into the mitochondria is primarily achieved through the diffusion of Ca^2+^ across the outer mitochondrial membrane (OMM) via the voltage-dependent anion channel (VDAC), and across the inner mitochondrial membrane (IMM) via the ruthenium red (RuR)-sensitive mitochondrial Ca^2+^ uniporter (MCU), and is driven by the electrochemical gradient. A puzzling observation was that the mitochondria took Ca^2+^ with an amplitude much higher than the predicted one based on the low affinity of MCU (Kd 15–20 µM). This was solved by the observation that some mitochondria are located in close apposition with Ca^2+^ release site of ER. Therefore, the release of high Ca^2+^ concentrations at contact sites between the two organelles leads to the formation of microdomains of high Ca^2+^ concentration that are crucial for efficient Ca^2+^ uptake by mitochondria^30^. The multiprotein complex involved in organelle Ca^2+^ transfer is composed of the inositol-1,4,5-triphoaphate receptor (IP3R) at ER membrane and of the VDAC at OMM, coupled by the chaperone glucose-regulated protein (Grp) 75^31^. Alterations of the interaction between ER and mitochondria lead to a disruption of Ca^2+^ transfer between ER and mitochondria and ER stress^32^. Therefore, MAMs are crucial for Ca^2+^ exchange between organelles. Then, Ca^2+^ accumulation into mitochondria is crucial for mitochondrial metabolism and energy production (see below) and for cell survival processes. When MAMs are disrupted, the release of Ca^2+^ from the ER mediated by IP3R, is suppressed and adenosine triphosphate (ATP) production and cell survival are reduced^33^. At the opposite, massive and/or a prolonged accumulation of Ca^2+^ into the mitochondria can lead to the opening of the permeability transition pore (PTP), swelling of the organelle, and the induction of apoptosis^34^.

#### ROS transfer

Beyond Ca^2+^, MAMs also support ROS-mediated signals that influence mitochondria function. Indeed, not only mitochondria generate ROS through the electron transport chain, but also the oxidative folding machinery in the ER produces H_2_O_2_ and includes ER oxidoreductin 1, which is localized to the MAMs^35,36^. ROS can change the activity of both ER and mitochondrial Ca^2+^ transport mechanisms, and ROS production itself is also affected by Ca^2+^^37^. Importantly, loss of the protein kinase RNA-like ER kinase (PERK) disrupted ER-mitochondria interactions and reduced ROS transfer from ER to mitochondria, protecting cells from ROS-mediated mitochondrial apoptosis^38^. Furthermore, the group of G. Hajnoczky recently employed drug-inducible synthetic ER-mitochondrial linkers and elegantly demonstrated that MAM interface hosted a nanodomain of H_2_O_2_, originated from the mitochondrial cristae, which is induced by cytoplasmic Ca^2+^ spikes and exerts a positive feedback on Ca^2+^ oscillations^39^.

### Metabolic interplay

#### MAMs and mitochondria biology

**Fig. 2:**
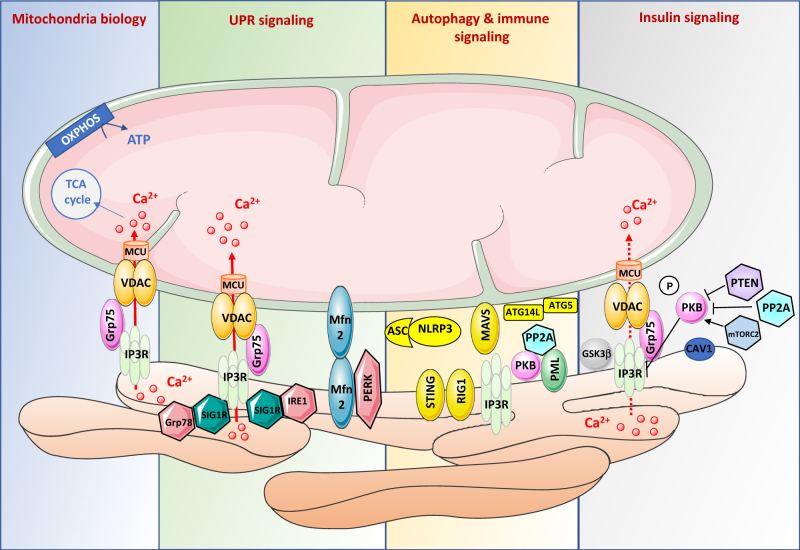
Key components and functions of MAMs involved in the control of glucose homeostasis. ER-mitochondria contact sites shelter several components that impact glucose homeostasis either indirectly by regulating mitochondria biology, UPR signaling and autophagy and immune signalling, or more directly by controlling insulin signaling

MAMs are now considered as structural platform for an optimal bioenergetics response allowing cellular adaptations to environmental changes. Indeed, the transfer of Ca^2+^ from ER to mitochondria is crucial for the control of mitochondria energy metabolism, since mitochondrial Ca^2+^ levels control the activity of Krebs cycle’s deshydrogenases and impact ATP synthesis^40^ (Fig. 2). In agreement, increased ER-mitochondrial coupling was shown to promote mitochondrial respiration and bioenergetics during early phases of ER stress^41^. However, sustained activation of ER stress impaired mitochondrial metabolism^41^, suggesting a strong link between metabolic insufficiency and ER stress-mediated apoptosis. In addition, genetic or pharmacological inhibition of IP3R altered mitochondrial function, lowering ATP production and triggering autophagy^42^. Thus IP3R-mediated Ca^2+^ release is important for cellular bioenergetics.

Beyond mitochondria bioenergetics, other aspects of mitochondria function are controlled by ER-mitochondria interactions, including mitochondria dynamics, apoptosis, and anti-viral signalling, each of which may influence glucose homeostasis. Most of these functions are intimately connected to the Ca^2+^ status of mitochondria, since IP3R-mediated Ca^2+^ transfer from ER to mitochondria control the activity of the dynamin-related protein 1 (Drp1)^43^, apoptosis^30^, and NOD-like receptor family, pyrin domain containing 3 (NLRP3) inflammasome activation^44^. Lastly, ER-mitochondria contacts were recently shown to coordinate the licensing of mitochondrial DNA (mtDNA) replication with division to distribute newly replicated nucleoids to daughter mitochondria, highlighting a new role of MAMs in mitochondria physiology^45^. However, its involvement in the control of glucose homeostasis is currently unknown.

#### MAMs and ER stress

The potential relationship between MAMs and the unfolded protein response (UPR) signalling (Fig. 2) was first suggested by the observation that loss of the MAM protein, phosphofurin acidic cluster sorting protein 2 (PACS2), disrupted ER-mitochondria interactions and induced ER stress^32^. In agreement, loss of other MAM proteins, such as Sigma receptor-1 (SigR1)^46^, mitofusin 2 (Mfn2)^47^, or cyclophilin D (CypD)^48^, also disrupted ER-mitochondria interactions and induced ER stress. Conversely, it was demonstrated that early phases of ER stress increased ER-mitochondria coupling to promote mitochondrial respiration and bioenergetics, whereas massive and/or prolonged mitochondrial Ca^2+^ accumulation induced swelling and dysfunction of the organelles^41^. PERK, an important ER stress sensor, is localized at MAMs and increase the physical coupling between both organelles^38^. Furthermore, PERK activity is regulated by Mfn2^49^, an important tether at MAM interface^50^, which have been associated with ER stress responses^47^. Lastly, the ER protein SigR1 also forms a complex with Grp78, another protein of UPR. Upon ER Ca^2+^ depletion, dissociation of this complex was shown to prolong Ca^2+^ signalling from the ER to mitochondria via IP3R at MAMs^46^. Increasing Sig1R expression in cells counteracts ER stress, thus inhibiting apoptosis^46^. As UPR signalling controls insulin action^9^ and secretion^10^, MAMs could therefore regulate glucose homeostasis through its effects on UPR signalling.

#### MAMs and autophagy

Autophagy is a cellular catabolic process degrading cellular constituents in order to generate energy in period of scarcity^51^, whereas mitophagy is a specific process to remove dysfunctional or superfluous mitochondria through the autophagy pathway^52^. Therefore, both autophagy and mitophagy may play an important regulatory role in glucose homeostasis. An involvement of MAMs in autophagy (Fig. 2) was firstly described, with the study of Hamasaki et al. showing that the autophagosome formation occurs at ER-mitochondria contact sites^53^. Indeed, the authors demonstrated that starvation induced an enrichment of different proteins of autophagy (ATG14L and ATG5) at MAM interface^53^. In agreement, disruption of MAMs by reducing PACS2 or Mfn2 expression decreased the number of autophagosomes, confirming that MAM integrity is a requirement for autophagasome formation. According to this model, disruption of MAMs by loss of Mfn2 inhibited the PS transfer from ER into mitochondria and starvation-induced autophagy^54^. Recent data further suggest that the VAPB-PTPIP51 tethers at MAMs is involved in the regulation of autophagy, by facilitating ER-mitochondria Ca^2+^ exchange, revealing a new molecular mechanism for regulating autophagy^55^. Very recently, a role of MAMs was further suggested in mitophagy. Gelmetti et al.^56^ have shown that following mitophagic stimuli, autophagosomes also form at MAMs, where endogenous PTEN-induced putative kinase 1 and beclin−1 relocalize and promote the enhancement of ER-mitochondria contact sites. In addition, the OMM FUN14 domain containing protein 1, which is involved in hypoxia-induced mitophagy^57^, has been also found to be enriched in MAMs and function as an adaptor protein coordinating mitochondrial dynamics and quality control^58^. Therefore, it is tempting to speculate that MAMs could regulate mitophagy in an adaptive fashion in order to adapt cellular metabolism to environmental changes.

#### MAMs and inflammation/immunity

Metabolic regulations are tightly coupled with inflammation and immune responses and exacerbated inflammatory responses have been linked to metabolic diseases^59^. ER-mitochondria contact sites were recently found to be an important actor of the cellular anti-viral response (Fig. 2). Indeed, several proteins involved in immune response to DNA viruses, such as the mitochondrial antiviral signalling protein (MAVS)^60^ or the stimulator of interferon genes^61^ were shown to be localized at MAM interface. Following viral infection, retinoic acid-inducible gene 1 is recruited at MAMs where it binds to MAVS in order to initiate a signalling cascade leading to the up-regulation of pro-inflammatory cytokines. This process is supported by the ER-mitochondria tethering function of Mfn2^60^. Interestingly, the hepatitis C virus NS3/4A protease, involved in the cleavage of MAVS to inhibit a strong antiviral response, was shown to target MAMs^60^, highlighting the role of MAMs in the regulation of innate immune signalling. Furthermore, MAMs are also involved in the formation and regulation of the inflammasome. Indeed, upon the activation of the NLRP3 and its adaptor, the apoptosis-associated speck-like protein, co-localized to the MAM interface^62^, suggesting that MAMs are important for the activation of a signalling pathway that leads to the processing and release of the pro-inflammatory cytokines.

#### MAMs and insulin signalling

A direct role of ER-mitochondria contact sites in the control of insulin signalling pathway was recently suggested, supporting the role of MAMs in the control of glucose homeostasis. Indeed, several proteins of insulin signalling were shown to be located at MAM interface, including the protein kinase Akt (also called PKB), the protein phosphatase 2A (PP2A), the mammalian target of rapamycin complex 2 (mTORC2), the phosphatase and tensin homolog (PTEN), and the glycogen synthase kinase 3β (GSK3β) (Fig. 2). Akt is present at MAMs^15,63,64^ where it phosphorylates IP3R, thus reducing Ca^2+^ release and preventing apoptosis^63^. The presence of Akt at MAMs seems to regulate MAM integrity since ER-mitochondria interactions are reduced in Akt KO cells^64^. Importantly, insulin stimulation increases Akt phosphorylation at MAM interface in the liver^15^, but it is actually unclear whether Akt is phosphorylated directly at MAMs or localized at MAMs after its phosphorylation in the cytoplasm. The activity of Akt at MAM interface is controlled by PP2A, which is also present in MAM fractions^63^. The dephosphorylation of Akt by PP2A counteracts its inhibitory action on IP3R, thus increasing Ca^2+^ transfer from ER to mitochondria^63^. Furthermore, mTORC2, a kinase phosphorylating and activating Akt^65^, is also present at MAM interface, where its presence increases in response to growth factors stimulation^64^. Interestingly, mTORC2 at MAMs controls Akt and its targets to ultimately control MAM integrity, Ca^2+^ release, and mitochondrial physiology^64^. In addition, the tumor suppressor PTEN is also enriched in MAMs, where it sensitizes cells to apoptosis by counteracting Akt-mediated phosphorylation and inhibition of IP3R, thus restoring ER Ca^2+^ release^66^. Lastly, a fraction of GSK3β was localized to MAMs in mouse heart, and was shown to interact with and activate IP3R in both adult cardiomyocytes and H9c2 cells, thus regulating organelle Ca^2+^ exchange^67^. Altogether, these data clearly indicate that MAMs are an important hub for insulin signalling that they may impact glucose homeostasis. In agreement, our laboratory recently demonstrated that MAM integrity is required for insulin signalling in both the liver^15^ and skeletal muscle^17^ and that MAM disruption is associated with hepatic^15^ and muscle^17^ insulin resistance. In agreement, mitochondrial Ca^2+^ uptake is critical for effective insulin signalling in both skeletal myocytes^68^ and cardiomyocytes^69^. Alternatively, cellular KO of key proteins of insulin signalling, such as Akt or mTORC2^64^, or disruption of insulin signalling by palmitate treatment^15,17^, disrupted MAM integrity, suggesting that the relationship between MAM integrity and insulin signalling is reciprocal. Whether MAMs control the canonical insulin signalling pathways in the cytosol or whether insulin signalling needs to transit by MAM interface to regulate metabolism should be determined in the future.

#### MAMs and glucose sensing

**Fig. 3:**
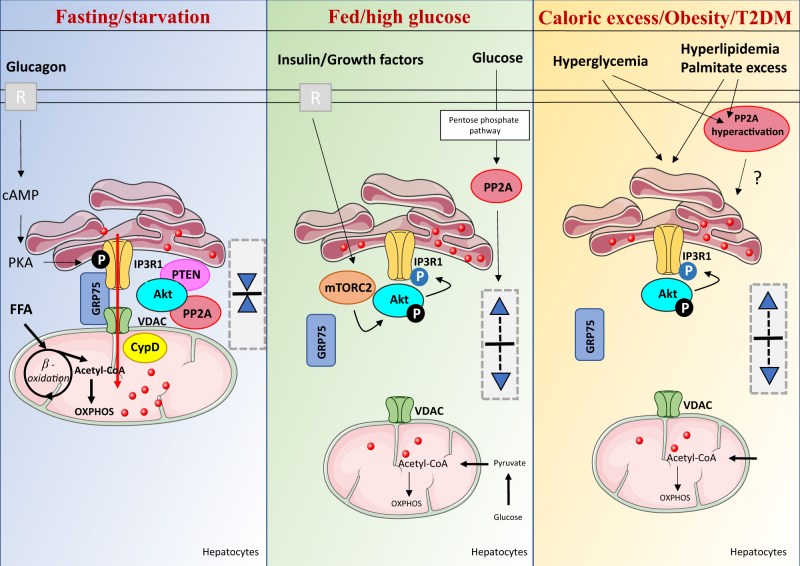
Dynamic regulations of ER-mitochondria interactions in hepatocytes in function of nutritional and pathological states. Left: At fasted or starved state, physical ER-mitochondria interactions are favored in order to increase oxidative capacities of mitochondria and preferentially use free fatty acids (FFA) as metabolic substrate. In this context, glucagon increases the phosphorylation and the activity of IP3R, leading to increased Ca^2+^ transfer from ER to mitochondria and to the stimulation of mitochondria metabolism. Both PP2A and PTEN probably counteract Akt-mediated reduction of IP3R-mediated Ca^2+^ release. Middle: At fed state or following high glucose stimulation, ER-mitochondria interactions and Ca^2+^ transfer are reduced by a mechanism dependent of PP2A. In this context, mTORC2 activate Akt phosphorylation, which induces the phosphorylation and inhibition of IP3R, leading to a reduction IP3R-mediated Ca^2+^ release. Upon caloric excess, increased glycemia or lipidemia reduce ER-mitochondria interactions. In this context, PP2A hyperactivation might participate to ER-mitochondria miscommunication associated with obesity and T2DM

Whereas both ER^70^ and mitochondria^71^ were independently considered as nutrient sensors allowing adaptation of cellular metabolism, MAM interface was recently further pointed as a nutrient-regulated hub that adapt mitochondrial metabolism to nutritional state^22^. As discussed above, the fact that disruption of MAMs inhibits starvation-induced autophagy^54^, that autophagosomes form at MAMs, and that different proteins of autophagy were enriched at MAMs after starvation^53^, initially suggested an important role of ER-mitochondria communication in starvation-induced processes. Next, ER-mitochondria interactions were further shown to double in length in the liver when nutrients become limiting^72^, suggesting that the liver could adapt to metabolic transitions through a mechanism dependent of MAMs. In agreement, our laboratory recently demonstrated that MAM integrity is regulated in liver during nutritional transition, with a reduction of ER-mitochondria interactions after feeding (Fig. 3)^16^. Importantly, glucose levels appear the major regulator of MAM integrity during nutritional transition, as increasing glucose levels can reproduce the effects of feeding on MAMs both in vitro and in vivo^16^. At the molecular level, we revealed that high glucose levels disrupted MAM integrity and function through the activation of the pentose phosphate -PP2A pathway^16^. Lastly, we demonstrated that the glucose-sensing by MAMs is crucial for the regulation of mitochondrial dynamics and function in the liver, since glucose-mediated reduction of MAMs induced mitochondria fission and impaired respiration^16^. Altogether, these data point to MAMs as a glucose sensor adapting to cellular bioenergetics, likely contributing to the adaptive fuel partitioning during nutritional transition^22^. Therefore, the capacity of MAMs to connect energy sensing to mitochondria physiology could be important for the control of glucose homeostasis.

## ER-mitochondria miscommunication in metabolic diseases

**Fig. 4:**
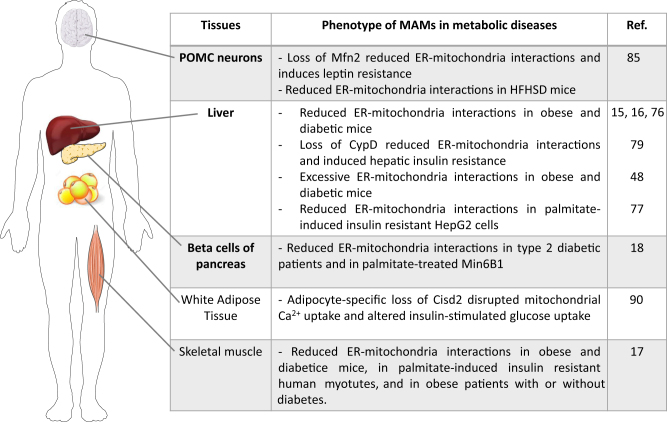
ER-mitochondria miscommunication in metabolic diseases. Listing of the different studies reporting ER-mitochondria miscommunication in different tissues involved in the control of glucose homeostasis

As MAMs are at the crossroad of several important hormonal and nutrient-regulated signalling pathways (see above), ER-mitochondria miscommunication may participate to metabolic diseases (Fig. 3). The discovery of ER-mitochondria miscommunication in hepatic insulin resistance has pushed MAMs into the spotlight of metabolic diseases^15^. While the field is evolving rapidly and controversies have emerged^73^, the role of MAMs in other insulin-sensitive tissues and in β-cells of pancreas has also been investigated. Today, an increasing array of data, further described below, confirm the key role of MAMs in the control of metabolic functions of different tissues involved in the control of glucose homeostasis (Fig. 4). Therefore, MAMs could be a new intracellular target to improve both insulin action and secretion and therefore improve more efficiently disrupted glucose homeostasis in the context of metabolic diseases.

### MAM alterations and hepatic insulin resistance

Whereas a strong interplay between mitochondria dysfunction and ER stress in the development of hepatic insulin resistance was initially suggested by different studies^47,74^, the specific involvement of ER-mitochondria miscommunication in hepatic insulin resistance was recently investigated by two independent groups, with conflicting results.

By using subcellular fractionation, in situ proximity ligation assay (PLA)^75^, and transmission electronic microscopy (TEM) analysis, our laboratory found that MAM integrity is disrupted in liver of different models of obese and diabetic mice (*ob/ob* and high-fat diet (HFD)-fed mice), as well as in palmitate-induced insulin resistant hepatocytes^15,16^. Importantly, disruption of MAM integrity by genetic or pharmacological inhibition of CypD induced insulin resistance in mice and disrupted insulin signalling in human primary hepatocytes^15^, confirming that MAM disruption is sufficient to induce hepatic insulin resistance and altered glucose homeostasis in vivo. Interestingly, treatment of diabetic mice with antidiabetic drugs (HFD mice with rosiglitazone or CypD-KO mice with metformin) improved insulin sensitivity and restored organelle communication^15^. In agreement, treatment of obese and diabetic mice with sulforaphane, a new potential anti-diabetic compound, improves disrupted ER-mitochondria interactions and suppresses exaggerated hepatic glucose production^76^. Conversely, the rescue of MAM integrity in primary hepatocytes of *ob/ob* or HFD mice by adenoviral overexpression of CypD improved insulin action^15^. Altogether, these data clearly demonstrate a strong relationship between disrupted MAMs and hepatic insulin resistance. This was confirmed by an independent group showing that disruption of MAMs is associated with palmitate-induced insulin resistance in HepG2 cells^77^. Mechanistically, we suggest that a loss of Ca^2+^ transfer from ER to mitochondria links MAM disruption to hepatic insulin resistance, at least in the liver of CypD-KO mice^48^.

Conversely, using TEM and co-labelling experiments, the group of G. Hotamisligil reported that MAM content is increased in the liver of genetically and diet-induced obese and diabetic mice, leading to mitochondrial Ca^2+^ overload, mitochondrial dysfunction and increased oxidative stress^73^. They further showed that reinforcing hepatic MAMs by IP3R1 or PACS2 overexpression induced oxidative stress and insulin resistance, whereas reducing the expression of these proteins in liver of obese mice improved mitochondrial oxidative capacity and insulin sensitivity in obese animals^73^. Altogether, these data established chronic enrichment of ER-mitochondria interactions as an essential component of organelle dysfunction in obesity that may contribute to hepatic insulin resistance.

The discrepancy between both studies is actually unclear but could be related to different factors related to the dynamic aspect of MAMs: (i) differences in mice metabolic status as MAM integrity is regulated by nutritional state^22^; (ii) differences in environmental housing conditions impacting microbiota flora and immune signalling since MAMs are sensitive to a variety of environmental signals, from nutrients to pathogens^78^; (iii) differences in experimental analysis since different methods exist to analyse MAM integrity and function and each ones has their limits^79,80^ or iv) differences in the investigated zones of the liver since the there is a metabolic zonation in the liver^81^. From my opinion, additive experimental parameters could explain the discrepancy between both studies, and each one needs to clarify some points. For example, in the study of Tubbs et al.^15^, organelle contact sites are mainly quantified by using *in situ* PLA experiments which do not take into account variations in mitochondria amount; therefore, the authors have to clarify whether in situ PLA experiments are influenced or not by mitochondria density. Furthermore, they need to investigate MAM functionality in their models to confirm that ER-mitochondria communication is really reduced with insulin resistance. In the study of Arruda et al.^73^, the authors also have to clarify whether their results are not influenced by experimental conditions, such as (i) the adenoviral infection of mice, which is a source of inflammation and ER stress and could influence ER-mitochondria interactions, (ii) the increased mitophagy, which could be easily be confused with increased MAMs in TEM analysis, or (iii) the increased MCU expression, which could be at the origin of the increase in Ca^2+^ transfer independently of variation in ER-mitochondria tethering, as suggested in another case of conflicting results related to Mfn2^50,80^. In addition, as none of MAM proteins are specific of this subcellular compartment, we cannot exclude that the modulation of their expression performed in both studies on different candidates could have non-specific effects, further participating to the discrepancy between studies. Lastly, it seems that several types of organelle contact sites exist^82^, with potentially different protein complexes and different function, and it is plausible that these different tethers could be differentially regulated, as recently suggested^83^. Indeed, the group of Tito Cali recently developed a split-GFP-based contact site sensor (SPICS) to follow narrow and wide organelle coupling, and showed that both sensors responded differently to several stimuli^83^. Therefore, we cannot exclude that narrow and wide organelle contact sites are differentially regulated with hepatic insulin resistance. Nevertheless, reduced or excessive ER-mitochondria contacts, likely depending on the timing of the adaptive response upon a metabolic challenge, could represent a new and important mechanism contributing to hepatic mitochondrial dysfunction and insulin resistance.

A disruption of ER-mitochondria interactions was also associated with insulin resistance in several knock-out mice models; however, the causal relationship between both parameters has not been investigated. For example, ER-mitochondria interactions were disrupted following cellular loss of mTORC2^64^, whereas mice with liver-specific KO of rictor, a mTORC2 subunit, showed impaired glucose and lipid homeostasis^84^. Similarly, loss of Mfn2 induced reduced ER mitochondria interactions in pro-opiomelanocortin (POMC) neurons^85^, whereas hepatic-specific loss of Mfn2 in mice induced hepatic insulin resistance and altered glucose homeostasis^47^. Furthermore, ER-mitochondria contacts are also reduced in POMC neurons of HFD mice^85^.

### MAM alterations and skeletal muscle insulin resistance

Skeletal muscle is another crucial tissue for glucose homeostasis as it is the primary site of insulin-stimulated glucose uptake and, therefore, the main target for alterations in insulin resistant states^86^. Interactions of mitochondria with the sarcoplasmic/endoplasmic reticulum (SR/ER) have been demonstrated in skeletal muscle^87^, but the role of organelle coupling in the control of muscle insulin action was unknown. We recently investigated this key question in different mouse models and humans^17^. We found a marked disruption of ER-mitochondria interactions in skeletal muscle of different mice models of obesity and type 2 diabetes, as an early event preceding mitochondrial dysfunction and insulin resistance. Furthermore, in human myotubes, palmitate-induced insulin resistance is associated with a reduction of structural and functional ER-mitochondria interactions. Experimental increase of ER-mitochondria contacts in human myotubes prevents palmitate-induced alterations of insulin signalling and action. Conversely, disruption of MAM integrity by silencing several endogenous MAM proteins or by overexpressing a testis-specific organelle uncoupler, namely FATE1^88^, alters the action of the hormone both in vitro and in vivo, indicating that reducing organelle coupling is sufficient to alter insulin signalling. Lastly, we found an association between altered insulin signalling and ER-mitochondria interactions in human myotubes from obese subjects with or without type 2 diabetes, compared to healthy lean subjects. Collectively, our data reveal the implication of MAMs in skeletal muscle insulin action and resistance in mice and humans^17^.

### MAM alterations and adipose tissue insulin resistance

Very few studies have investigated the relevance of MAMs in the metabolism and insulin sensitivity of WAT. One of them focuses on the new role of Cisd2 in the maintain of intracellular Ca^2+^ homeostasis, as well as in the regulation of glucose homeostasis in mice. It has been reported that Cisd2, a causative gene of Wolfram syndrome 2, is localized on both ER and mitochondrial membranes and at MAMs in various cell types^89^. At MAMs, Cisd2 interacts with Gimap5 and modulates mitochondrial Ca^2+^ uptake, in order to maintain intracellular Ca^2+^ homeostasis^90^. Interestingly, conventional loss of Cisd2 altered mitochondria function, impaired glucose tolerance and induced premature aging^89^, whereas WAT-specific loss of Cisd2 impaired the Ca^2+^ buffering capability of mitochondria, increased cytosolic Ca^2+^, impaired the differentiation of WAT, and decreased insulin-stimulated glucose transport^90^. Altogether, these data suggest that Cisd2-mediated loss of organelle coupling might be involved in WAT insulin resistance and altered glucose homeostasis. Further works are required to confirm this assumption.

### MAM alterations and β-cell dysfunction

Whereas several studies pointed an important role of disrupted Ca^2+^ homeostasis in beta cell dysfunction, very few studies have directly investigated the role of MAMs. Using fixed human pancreatic tissues obtained from brain-dead organ donors with or without diabetes, our laboratory recently demonstrated that ER-mitochondria interactions measured by *in situ* PLA are reduced in β-cells from type 2 diabetic patients compared to non-diabetic controls^18^. Interestingly, treatment of Min6-B1 cells with palmitate altered both glucose-stimulated insulin secretion and ER-mitochondria interactions^18^. Altogether, these data suggest a potential involvement of organelle interactions in the control of insulin secretion by β-cells. However, further studies are required to clearly demonstrate that the experimental modulation of MAMs regulate β-cell function, and to investigate the relevance of MAMs in β-cell dysfunction during T2DM.

### MAM alterations and hepatic metabolic inflexibility

T2DM is classically associated with metabolic inflexibility^91^. Importantly, we found that chronic disruption of MAMs in the liver of insulin resistant mice is associated with a loss of MAM regulation by change in nutritional state^16^. Indeed, fasting to post-prandial transition reduced ER-mitochondria interactions in liver of wt mice, whereas this regulation is lost in liver of obese and diabetic mice, characterized by chronic disruption of MAM integrity, mitochondrial fission and altered mitochondrial respiration^16^. Therefore, chronic disruption of MAMs may participate to both hepatic metabolic inflexibility and mitochondrial dysfunction associated with hepatic insulin resistance. In line with these evidences, ER-mitochondria interactions are controlled by PP2A^16^ and hyperactivation of PP2A was associated with insulin resistance^92^. Therefore, increased PP2A activity could participate to disruption of MAMs in liver of insulin-resistant mice. Future studies are required to understand the molecular mechanisms of MAM disruption in the context of hepatic metabolic diseases.

## Conclusions and perspectives

It is now clear that MAMs are at the crossroad of several important hormonal and nutrient-regulated signalling pathways in metabolic tissues, suggesting that ER-mitochondria miscommunication could be involved in metabolic diseases. However, the few reported studies on this topic are rather controversial. Therefore, it is now crucial that the next studies solve this controversy. In any case, targeting MAMs might be a novel strategy for the treatment of T2DM, especially if ER-miscommunication is also involved in β-cell dysfunction. For that we need now to more understand the physiological regulations of ER-mitochondria interactions in order to identify key intracellular targets to improve organelle crosstalk. Currently, we only know that some hormones^15,17^ and growth factors^64^, as well as some nutrients^16^ can regulate organelle proximity, but the molecular mechanisms are unknown. Finally, the results described until now are mainly obtained in mice models or in cultured cells, therefore further studies in humans are required. There is no doubt that these important questions will be shortly resolved and will clarify the knowledge of MAMs in metabolic health and diseases.
